# *Alstonia scholaris* R. Br. Significantly Inhibits Retinoid-Induced Skin Irritation *In Vitro* and *In Vivo*

**DOI:** 10.1155/2012/190370

**Published:** 2011-09-08

**Authors:** Soo-Jin Lee, Sun-A Cho, Su-Sun An, Yong-Joo Na, Nok-Hyun Park, Han-Sung Kim, Chan-Woo Lee, Han-Kon Kim, Eun-Kyung Kim, Young-Pyo Jang, Jin-Woong Kim

**Affiliations:** ^1^Amorepacific Co. R&D Center, Bora-dong, Giheung-gu, Yongin-si, Gyeonggi-do 449-729, Republic of Korea; ^2^College of Pharmacy, Kyung Hee University, Hoegi-dong, Dongdaemun-gu, Seoul 130-701, Republic of Korea; ^3^Department of Applied Chemistry, Hanyang University, 55 Hanyangdaehak-ro, Sangnok-gu, Ansan, Gyeonggi-do 426-791, Republic of Korea

## Abstract

Topical retinoids inhibit matrix metalloproteinases and accelerate collagen synthesis, thereby triggering antiaging effects in the skin. However, topical retinoids can cause severe skin reactions, including scaling, erythema, papules, and inflammation. The present study demonstrates that the ethanolic bark extract of *Alstonia scholaris* R. Br. can significantly inhibit *all-trans* retinoic acid-induced inflammation in human HaCat keratinocyte cells. Furthermore, two representative retinoid-induced proinflammatory cytokines, monocyte chemoattractant protein-1 and interleukin-8, were significantly suppressed by *A. scholaris* extract (by 82.1% and 26.3% at 100 ppm, and dose-dependently across the tested concentrations) *in vitro*. In a cumulative irritation patch test, *A. scholaris* extract decreased retinol-induced skin irritation, while strengthening the ability of retinoids to inhibit matrix metalloproteinase-1 expression, which is strongly associated with aging effects. These results suggest that *A. scholaris* is a promising compound that may increase the antiaging function of retinoids while reducing their ability to cause skin irritation.

## 1. Introduction

Skin, which is the largest organ of the human body, functions as a physical, biological, and physiological barrier. These functions can be impaired by external and internal factors, such as ultraviolet (UV) light, xenobiotics, and hormonal changes [1–3]. These factors trigger various signs of skin aging, which is commonly characterized by the formation of fine wrinkles, reduced water content, and decreased skin thickness [2]. Wrinkling or photodamaging of the upper dermis is closely associated with disorganization of collagen/elastin-based connective tissues [4], while wrinkles are formed by degradation of the extracellular matrix via changes in matrix metalloproteinase (MMP) levels [5, 6]. The involved MMPs include several members of the zinc endopeptidase family: collagenase-1 (MMP-1), stromelysin (MMP-3), and 92-kDa gelatinase (MMP-9) [7, 8]. 

 The topical application of retinoids relieves skin wrinkles caused either by natural aging [2] or photoaging [7, 9–11]. Furthermore, retinoids are widely used as topical treatments for various conditions, such as acne and psoriasis, and in dermatology clinics for skin cancer therapy [9, 12, 13]. Topical retinoids inhibit the UV-induced, MMP-mediated breakdown of collagen [8] and protect against UV-induced decreases in procollagen expression [14]. However, topical retinoid therapy is frequently accompanied by inflammation; this is commonly known as “retinoid dermatitis” [15–17]. Currently, scientists are seeking to overcome the problem of retinoid dermatitis. Some approaches using novel ingredients have been proposed, but it would be very useful to develop anti-irritants capable of reducing the disadvantages of topical retinoid therapy.

In certain individuals, they experience more intense and frequent adverse sensory effects than the normal population after topical use of personal care products, a phenomenon known in popular usage as sensitive skin [18]. A recent report showed that approximately 40% of people consider themselves to possess the characteristics of sensitive skin [19], which represents as a skin type showing higher reactivity than normal skin and developing exaggerated reactions when exposed to external factors [20]. It is a complex problem with genetic, individual, environmental, occupational, and ethnic implications, and subjective symptoms of sensitive skin include erythema, itching, burning, and stinging [20, 21], which are often closely related to abnormal skin barrier function and/or accelerated nerve responses. Peripheral activation of certain afferent sensory neurons has been shown to produce various inflammatory responses. Cutaneous nerve fibers are important regulators in this process, which is called “neurogenic inflammation” [22]. They contain proinflammatory neuropeptides, such as calcitonin gene-related peptide (CGRP), vasoactive intestinal peptide (VIP), and tachykinin-like substance P (SP) [23]. Among these, CGRP is a 37-amino acid peptide known to be a major component released from nerve endings [24]. 

 Folk medicine has a long history of treating diverse diseases, and some herbal folk medicines have been adopted by the pharmaceutical and cosmetic industries. Here, we assess the potential use of *Alstonia scholaris* (L.) R. Br. as a novel anti-irritant for reducing retinoid-induced dermatitis. *A. scholaris *is a tree belonging to the family Apocynaceae; it is widely distributed in South and Southeast Asia [25] and has traditionally been used to treat asthma, pneumonia, and fever [26]. Despite this wide use, no prior study that we are aware of has examined the potential anti-inflammatory or anti-irritative activities of *A. scholaris* in skin. Here, we prepared an alcoholic extract from the stem bark of *A. scholaris* and investigated its potential anti-inflammatory effects on human skin.

## 2. Material and Methods

### 2.1. Plant Materials and Extraction

*A. scholaris *was kindly supplied by the Institute of Natural Products Chemistry (Vietnam). A voucher specimen (KHUP-0107) was deposited in the Museum of Korean Crude Drugs (Kyung Hee University, South Korea). Dried stem bark of *A. scholaris* (190 g) was extracted with 70% ethanol (2 L × 3) at room temperature using a sonicator (3 h × 3). The extract was evaporated to dryness under a vacuum at 40°C, yielding a brown residue (31.5 g).

### 2.2. Cell Culture

Human HaCaT keratinocytes were cultured in Dulbecco's modified Eagle's medium (DMEM, Lonza, Basel, Switzerland) supplemented with 10% heat-inactivated fetal bovine serum (FBS; Gibco, Carlsbad, Calif, USA) and 100 U/mL penicillin/streptomycin (Lonza), at 37°C in a humidified atmosphere containing 5% CO_2_. Primary dermal fibroblasts were obtained from human adult foreskins obtained from healthy volunteers and cultured in DMEM supplemented with 1% penicillin/streptomycin and 10% heat-inactivated FBS at 37°C in a humidified atmosphere containing 5% CO_2_. The cells were cultured to 90% confluence before being passaged; passages 4 to 7 were used for experiments. 

### 2.3. Cell Viability Assay

Cell viability tests were performed for cultures exposed to the following agents: *all-trans* retinoic acid (ATRA; Sigma-Aldrich, St. Louis, Mo, USA), retinol (ROL; Sigma-Aldrich), ASE (*Alstonia scholaris* extract), and mixtures of ATRA/ASE and ROL/ASE. 

### 2.4. *In Vitro* Assay for Inhibition of ATRA-Induced Inflammation

HaCaT cells were seeded in a 96-well plate (2.0 × 10^4^ cells/well) and grown for 24 h. The resulting monolayers were washed three times with 200 *μ*L of phosphate buffered saline (PBS; Lonza), and then 200 *μ*L of serum-free DMEM was added to each well. After 24 h of serum starvation, the monolayers were washed three times with PBS, and 150   *μ*L of 1% FBS-containing media was added. Cells were treated with 50   *μ*L of diluted anti-irritants (madecassoside or hydrocortisone) or ASE for 10 min, then 50 *μ*L of 1 *μ*M ATRA in 1% FBS-containing media was added to the anti-irritant-treated cells, and incubation was continued for another 24 h. Supernatants were collected, and enzyme-linked immunosorbent assay (ELISA) was performed against interleukin-8 (IL-8) and monocyte chemoattractant protein-1 (MCP-1) (BD OptEIATM; BD Biosciences, San Diego, Calif, USA) according to the manufacturer's protocol. Each conjugate was incubated with 2,2′-azino-bis-3-ethylbenzthiazoline-6-sulfonic acid (Sigma-Aldrich), and absorbance was measured at 405 nm using a SoftMax Pro 5 (Molecular Devices, Sunnyvale, Calif, USA). 

 All samples were tested in triplicate, and the protein amounts were expressed in pg/mL. The reduction % of ATRA-induced irritation was calculated using the following formula: reduction % of irritation = (cytokine protein level in ATRA-treated group − cytokine protein level in anti-irritant treated group)/(cytokine protein level in ATRA-treated group − cytokine protein level in nontreated control group) × 100. The experimental data were expressed as averages ± SD, and significance was analyzed using the two-sample *t*-test (Minitab 14.0; Pennsylvania, Minitab Inc.), with *P* < 0.05  considered statistically significant.

### 2.5. *In Vitro* Assay for Inhibition of ATRA-Induced MMP-1 Expression

Primary fibroblasts were seeded in a 2-well plate (3.0 × 10^4^ cells/well) and grown for 24 h. The resulting monolayers were washed three times with 2 mL of PBS, and then 2 mL of serum-free/phenol red-free DMEM was added to each well. After 24 h, the serum-starved cells were washed three times with PBS and exposed to UVB irradiation (UVB lamp, G15TBE, Sankyo Denki, Japan); the total energy dose of UVB irradiation was 40 mJ/cm^2^. After UVB exposure, serum-free DMEM containing the indicated concentrations of ATRA, ROL, ASE, ATRA/ASE, or ROL/ASE was added to the cells and incubation was continued for an additional 24 h. Supernatants were collected and subjected to MMP-1-specific sandwich ELISA (RPN 2610, Amersham Bioscience, Buckinghamshire, UK) according to the manufacturer's protocols, using precoated 96-well immunoplates, rabbit antihuman MMP-1 antibodies, horseradish-peroxidase-conjugated antirabbit IgG, and 3,3′,5,5′-tetramethyl benzidine (TMB, used as a peroxidase substrate; Sigma-Aldrich). Absorbance was read at 450 nm using a SoftMax Pro 5.0 (Molecular Devices). 

All samples were tested in triplicate and the proteins amounts were expressed in ng/mL. The inhibition % of MMP-1 expression was calculated by following formula: Inhibition % of MMP-1 expression = (MMP-1 protein level in the UVB-irradiated group – MMP-1 protein level in the test-material-treated group)/(MMP-1 protein level in the UVB-irradiated group − MMP-1 protein level in the non-treated control group) × 100. The experimental data were expressed as averages ± SD and significance was analyzed using the two-sample *t*-test (Minitab 14.0), with *P* < 0.05 considered statistically significant.

### 2.6. Neuronal Cell Culture and Calcitonin Gene-Related Peptide (CGRP) Assay 

Human SK-N-BE(2) and SH-SY5Y neuroblastoma cells (American Type Culture Collection, Manassas, Va, USA) were grown in DMEM supplemented with 2 mM/L L-glutamine, 10% heat-inactivated FBS, and 1% penicillin/Streptomycin (Invitrogen, Carlsbad, Calif, USA). To induce differentiation, cells were seeded on 12-well plates (5.0 × 10^4^  cells/well) in serum-containing medium and then stimulated for 2 weeks with 10 *μ*M ATRA, 10 *μ*M ATRA plus 1 ppm ASE, or 10 *μ*M ATRA plus 10 ppm ASE. The medium was replaced every 2-3 days. The differentiated SK-N-BE(2) and SH-SY5Y cells were then incubated in serum-free DMEM containing 300 nM capsaicin for 4 h, and the levels of CGRP in the conditioned media were determined by ELISA (E0876h; USCNLIFE, Missouri, Tex, USA). 

### 2.7. Anti-Inflammatory Effect on ROL-Induced Irritation in Human Skins

To evaluate the potential ability of ASE to decrease ROL-induced irritation in human skin, a modified cumulative irritation (mCI) test was performed. An oil-in-water (O/W) emulsion was prepared by homogenizing a 13.9 wt% oil mixture in water at 7.0 × 10^3^ rpm for 5 min at 70°C. The utilized oil mixture consisted of 6.5% cetearyl alcohol/cetearyl glucoside (MONTANOV 68, Seppic, France), 3.6% glyceryl stearate/PEG-100 stearate (ARLACEL 165VEG; Croda, Edison, NJ, USA), 3.6% glyceryl stearate, 7.2% cetearyl alcohol, 21.6% squalane, 21.6% cetyl ethylhexanoate, and 36% decamethyl cyclopentasiloxane. The viscosities of the emulsions were adjusted to ~4.0 × 10^4^ cps by tuning the concentration of CARBOPOL ETD 2020 polymer (Lubrizol Advanced Materials, Cleveland, Ohio, USA). A stabilized retinol system was used to encapsulate the retinol (0.075%, 2500 international units (IU)) in polymer particles, which were then homogeneously dispersed in the emulsion at room temperature [27].

 The resulting creams, one containing 2500 IU retinol (3.33 IU = 1 retinol equivalent (RE) = 1 *μ*g of retinol) with 0.1% ASE (test emulsion) and one containing 2500 IU retinol alone (control emulsion), were tested on 21 healthy adults (8 males and 13 females). For the tests, 30 *μ*L of cream was applied to a 2.5-cm-diameter area of the volar forearm and rubbed 50 times. The treated area was then wrapped with plastic wrap for 3 h. This procedure was performed twice each business day for 3 weeks (total 15 days. Every day prior to the first application, the skin reactions were graded according to a modified criteria proposed by Frosch and Kligman in 1979 [28] and CTFA (Cosmetic, Toiletry, and Fragrance Association) guidelines of 1981 as follows: 0 = no reaction; 1 = slight erythema, spotty or diffuse; 2 = moderate uniform erythema; 3 = intense erythema with edema; 4 = intense erythema with edema and vesicles. Various other skin symptoms (scales, fissures, etc.) were also noted. If a subject showed skin irritations over grade 3, the topical application was stopped. However, the skin examinations continued to the end of test. 

The numerical score (cumulative irritation index) of each subject was summed, and the mean was compared between the test emulsion (ROL + 0.1% ASE) and control emulsion (ROL). The maximum possible score was 1260 (21 persons × 15 days × 4). The irritation index was calculated as follows: average = sum/number of subjects (*n* = 21). Test results were statistically analyzed using the nonparametric Mann Whitney test (Minitab 14.0), with *P* values < 0.01 regarded as significant. The formulated emulsions were stored at 4°C during the test period. The retinol titer did not significantly differ between day 0 and day 15. This study was approved by the ethics committee of the DERMAPRO/Skin Research Center (Seoul, Republic of Korea), and patients gave written informed consent.

### 2.8. Human Repeat Insult Patch Test (HRIPT)

To evaluate the sensitization potential of ASE toward human skin, a repeat insult patch test (RIPT) was performed [29, 30]. We prepared an oil-in-water (O/W) emulsion by homogenizing a 18.8 wt% oil mixture in water at 7.0 × 10^3^ rpm for 5 min at 70°C. The oil mixture consisted of 26.6% hydrogenated polydecene, 26.6% cetyl octanoate, 26.6% limnanthes alba (meadowfoam) seed oil, 8% polysorbate 60, 5.3% cetearyl alcohol, 5.3% glyceryl stearate, and 1.6% sorbitan stearate. The viscosities of the emulsions were adjusted to ~4.0 × 10^4^ cps by tuning the concentration of sepiplus 400 (Seppic, France). 

Emulsions (20 *μ*L) with or without 0.2% ASE were applied to the volar forearms of volunteers using IQ chambers (Chemotechnique Diagnostics, Sweden). The patches were removed after 24 h, and skin responses were evaluated within 1 h of patch removal. Three induction patches were applied each week for a total of 3 weeks. Following a 2-week rest period (during which no patches were applied), a single challenge application of the same material was applied to a naïve site (another arm) and left on for 48 h. Skin responses were evaluated at 30 min, 24 h, and 48 h after patch removal. The skin responses obtained during the induction phase were scored according to the modified criteria proposed by Frosch and Kligman in 1979 [28] and the CTFA guidelines of 1981 [31]. During the challenge phase, the results were scored according to the terminology established by the International Contact Dermatitis Research Group (ICDRG). Briefly, the condition of the skin in the treated areas was classified as follows: 1 (+) = doubtful reaction consisting of only faint erythema; 2 (+) = weak positive reaction comprising erythema, infiltration, and possibly papules; 3 (++) = strong positive reaction comprising erythema, infiltration, papules, and vesicles; 4 (+++) = extreme positive reaction comprising intense erythema and infiltration, as well as coalescing vesicles. Various other skin symptoms (scales, fissures, etc.) were also noted. Each examination was performed under standard-light conditions by a qualified research expert or a dermatologist. This study was approved by the above-noted ethics committee, and patients gave written informed consent.

### 2.9. HPLC Analysis

Chromatographic measurements were performed using an HPLC system (Waters, Milford, Mass, USA) comprising a 515 pump, a 717 autosampler and a 996 photodiode array detector, and operated by the Waters Empower software. Chemical fingerprint analysis was performed using a Shiseido Capcell-pak C18 (Tokyo, Japan) column (250 × 4.6 mm i.d.; 5 *μ*m). The UV data were collected from 200 to 400 nm. The mobile phase comprised methanol (solvent A) and water (solvent B) in a linear gradient that increased from 5% solvent A to 100% A over 55 min. The flow rate was 0.8 mL/min, and the injection volume was 7 *μ*L.

### 2.10. Electrospray Ionization with Time-of-Flight Mass Spectrometry (ESI-TOFMS)

Mass spectra were measured by ESI-TOFMS. The ESI ion source (Jeol, Tokyo, Japan) was coupled to a JMS-T100TD (AccuTOF-TLC, Tokyo, Japan) in the positive-ion mode with a discharge needle voltage of 2000 V and a nebulizing nitrogen gas flow of 1.5 L/min. The first orifice lens was set to 100 V, and the ring lens was set to 13 V. The TOF-MS was set with a peak voltage of 2500 V, a bias voltage of 29 V, a pusher bias voltage of −0.76 V, and a detector voltage of 2300 V.

## 3. Result

### 3.1. HPLC Fingerprint of the *A. scholaris* Extract (ASE)

**Figure 1:**
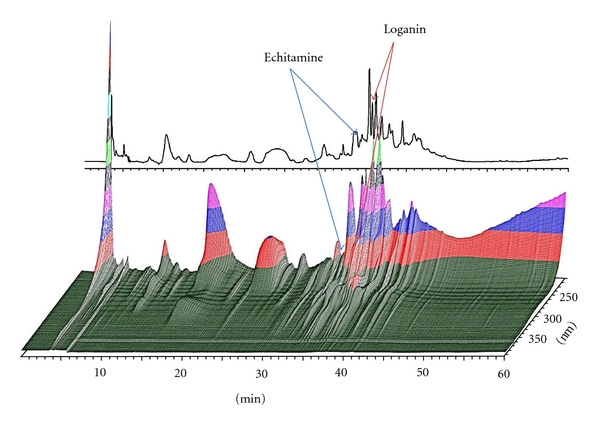
HPLC chromatogram of the ethanolic extract of *A. scholaris*.

To compare our generated ASE with results from previous papers and establish a standard chromatogram for future quality control of ASE, a standard HPLC chromatogram was performed and the results were corroborated across various column types and mobile phase compositions. A representative result is shown in Figure 1. Two compounds previously identified from this plant were well resolved from other components in the chromatogram [32, 33]. Their identities were confirmed by comparing the high-resolution mass spectra measured from the Accu-TOF analyzer with calculated values. The experimental mass value of the echitamine ion was 385.2127 (calc. 385.2086), while that of the sodiated adduct of the loganin ion was 413.1423 (calc. 413.1396). 

### 3.2. Anti-Inflammatory Effects of ASE on ATRA-Induced Inflammation *In Vitro*

**Figure 2:**
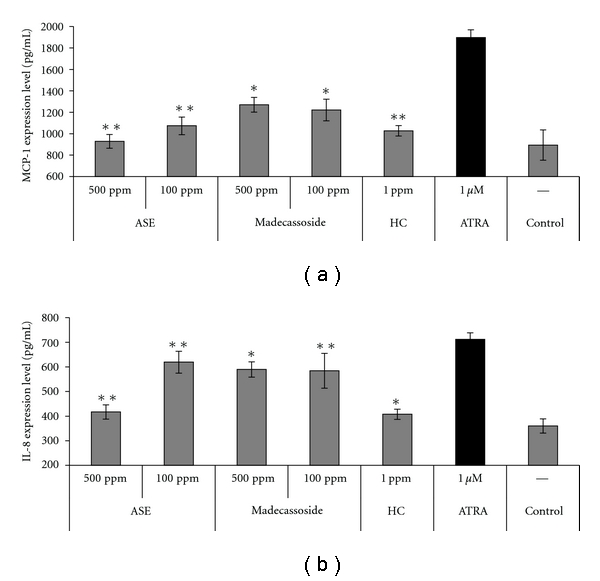
Anti-inflammatory effect of *A. scholaris* extract (ASE) on HaCaT human keratinocytes treated with *all-trans* retinoic acid (ATRA), as assessed by monitoring the protein expression of (a) monocyte chemoattractant protein-1 (MCP-1) and (b) interleukin-8 (IL-8). All results are expressed as average ± SD. *P < 0.05 and **P < 0.01 compared with the ATRA-treated group.

Next, we investigated the potential anti-inflammatory effects of ASE and compared them to those of two well-known compounds, madecassoside and hydrocortisone. HaCaT cells were treated with ATRA with or without the indicated concentrations of the various agents, and the level of proinflammatory cytokines, which are interleukin-8 (IL-8) and monocyte chemoattractant protein-1 (MCP-1), was measured in collected supernatants. As shown in Figure 2 and Table 1, ASE conferred a strong anti-inflammatory effect with ASE-treated cells showing MCP-1 levels that were 928.8 ± 64.0 pg/mL (at 100 ppm) and 1074.0 ± 82.2 pg/mL (at 500 ppm) lower than those in control cells. This inhibition was less than that seen in madecassoside-treated cells, in which the MCP-1 levels decreased by 1221.6 ± 100.8 pg/mL (at 100 ppm) and 1271.0 ± 69.0 pg/mL (at 500 ppm), but it was comparable to that seen in hydrocortisone-treated cells (1027.6 ± 48.5 pg/mL at 1 ppm). ASE also significantly decreased IL-8 expression by 619.3 ± 44.4 pg/mL at 100 ppm and 417.1 ± 29.0 pg/mL at 500 ppm; these were comparable to the results obtained from madecassoside, which decreased IL-8 expression by 584.6 ± 70.6 pg/mL at 100 ppm and 589.8 ± 31.0 pg/mL at 500 ppm. The effective concentrations of ASE were not cytotoxic to HaCaT cells (data not shown), indicating that the observed anti-inflammatory effects were not associated with cytotoxicity.

### 3.3. Inhibitory Effect of ASE on MMP-1 Production by Irradiated Human Dermal Fibroblasts

**Figure 3:**
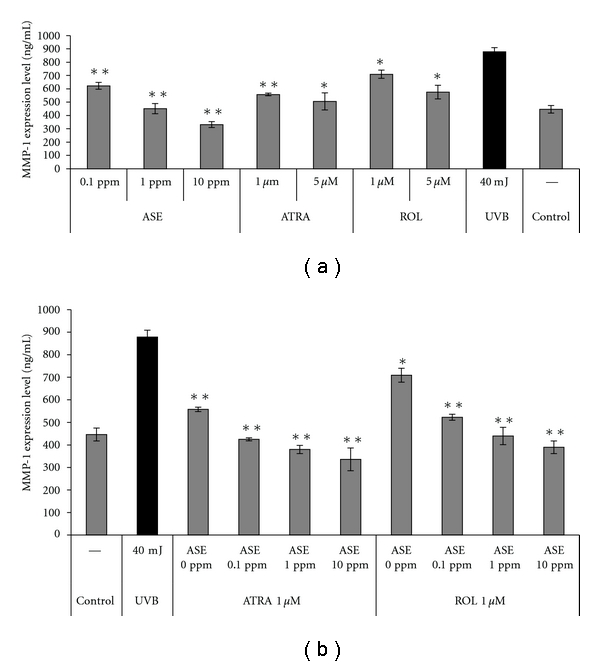
Effect of ASE associated with UV-induced MMP-1 expression on human primary fibroblasts. (a) Matrix metalloproteinase-1 expression in UVB-irradiated human primary fibroblasts treated with or without ASE. (b) Effect of ATRA or retinol (ROL) plus ASE on matrix metalloproteinase-1 expression. *P < 0.05 and **P < 0.01 compared with the UVB-irradiated group.

To further examine the effects of ASE, we examined the production of MMP-1 by UVB-irradiated human dermal fibroblasts in the presence and absence of ASE and whether ASE could affect photoaging. As shown in Table 2(a) and Figure 3(a), UVB-irradiated human dermal fibroblasts produced 2-fold more MMP-1 than nonirradiated control cells, but treatment of these cells with ATRA, ROL, and ASE effectively and dose-dependently inhibited MMP-1 production. Notably, cotreatment of cells with ASE/ATRA or ASE/ROL enhanced the inhibition effect (Table 2(b) and Figure 2(b)). Treatment with 10 ppm ASE plus 1 *μ*M ATRA or ROL significantly attenuated MMP-1 expression by 335.6 ± 50.5 ng/mL and 389.3 ± 28.1 ng/mL, respectively. These values were much lower than the inhibitions observed in cells treated with ATRA or ROL alone (557.4 ± 9.7 ng/mL for 1 *μ*M ATRA; 708.9 ± 30.8 ng/mL for 1 *μ*M ROL). This finding indicates that the use of ASE appears to have a synergistic effect on the retinoid-induced suppression of MMP-1 expression in this system. 

### 3.4. The Effect of ASE on Capsaicin-Induced CGRP Production

To further investigate the effect of ASE on neurogenic inflammation, we assessed the production of CGRP from differentiated neuronal cells treated with 300 nM of capsaicin with or without ASE. The results are summarized in Table 3. Briefly, CGRP production was increased about 150~250% by capsaicin treatment, but this increase was dose-dependently inhibited by cotreatment with ASE. We further observed that ASE did not affect neurite outgrowth compared with that in neuronal cells treated with capsaicin alone. 

### 3.5. Anti-Inflammatory Effect of ASE on Retinol-Induced Irritation of Human Skin

**Figure 4:**
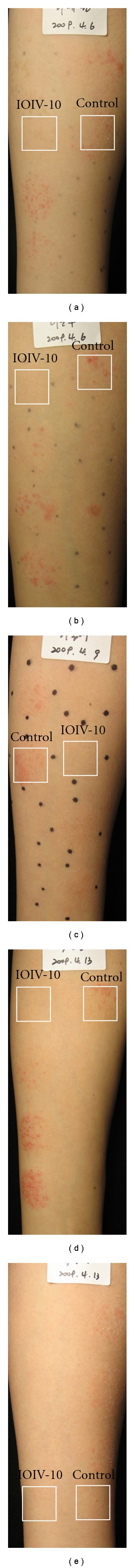
The pictures of five volunteer subjects: effect of ASE on retinol-induced skin irritation *in vivo*. IOIV-10: test emulsion containing 2500 IU ROL and 0.1% ASE; control: emulsion containing 2500 IU ROL.

The anti-irritation potential of ASE on human skin was evaluated by the cumulative irritation test using retinol-contained emulsion in the presence and the absence of ASE. The study population consisted of 21 subjects aged 23 to 36 (mean age of 28.2), and skin irritation responses were scored. The response tendency of several subjects are shown in Figure 4. Briefly, the mean irritation score of the test emulsion was 1.95, whereas that of the control emulsion (lacking ASE) was 5.33 (Table 4). Thus, the presence of ASE appeared to significantly decrease ROL-induced skin inflammation (by ~60%; *P* < 0.01).

### 3.6. *In Vivo* Sensitization Potential of ASE in Human Skin

To evaluate the sensitization potential of ASE toward human skin, HRIPT was carried out on 59 healthy female volunteers aged 18 to 48 (mean age 34.5); 54 volunteers completed the test, while five subjects discontinued for personal reasons. During the induction phase, 18 volunteers showed skin reactions of grade 1 to the test emulsion, nonetheless, they did not show any certain tendency or specificity concerned with cumulative skin irritation. 20 volunteers displayed skin reactions of grade 1 to the control emulsion, but these symptoms disappeared within 72 h and did not show any specificity. In the challenge phase, nine subjects who received the test emulsion showed skin reactions of grade 1+; these skin reactions disappeared within 72 h among eight of these patients, while one (#08) showed a skin reaction that was sustained to 96 h without any further signs. In order to check the reproducibility of this result, a second challenge was performed on subject #08. During the second challenge phase, this patient did not show any skin response (data not shown). Among the subjects who received the control emulsion, eight showed skin reactions of grade 1+, but these signs disappeared within 72 h. These HRIPT results suggest that ASE could trigger very weak, transient skin responses, but did not appear to induce allergy-specific symptoms (e.g., edema and itching) or any reproducible skin pathology. 

## 4. Discussion

The phytochemical constituents of *Alstonia spp.* have been extensively investigated, with nearly 400 compounds isolated and characterized to date [25, 34, 35]. In particular, ASE is known to comprise a variety of alkaloids, flavonoids, and terpenoids [25, 36, 37]. The constituent alkaloids include alstonidine, alstonine, chlorogenic acid, chlorogenine, ditain, echitamine, and echitenin, while the triterpenoids include lupeol linoleate and lupeol [38–40]. The major alkaloid obtained from the bark is echitamine, which may be isolated as a chloride [41]. In the present work, our Accu-TOF analysis identified two major compounds from ASE, echitamine and loganin (Figure 1), which are well-known for both their anti-inflammatory effects and their cough-relieving activities [42–44]. Therefore, the anti-inflammatory effects of *A. scholaris* identified herein may be due to the actions of its major components, echitamine and loganin, along with other compounds, such as flavonoids and terpenes. 

We then evaluated the ability of ASE to inhibit retinoid-induced inflammation of the human skin. Retinoid-induced dermatitis is typically characterized by mild erythema, peeling of the stratum corneum, and manifestation of various other symptoms that are mediated by inflammatory cytokines, such as monocyte chemoattractant protein-1 (MCP-1) and interleukin-8 (IL-8) [45]. Our results revealed that ASE dose-dependently down-regulated the ATRA-induced releases of MCP-1 and IL-8 from HaCaT human keratinocytes, indicating that the extract can dramatically inhibit the ATRA-induced secretion of inflammatory cytokines (Figure 2). The inhibitory effects of ASE were higher than that of madecassoside at 500 ppm, a well-known natural anti-inflammatory compound whose major active compound is the pentacyclic triterpenoid saponin isolated from *Centella asiatica* [46–48].

Increases in MMP-1 activity have been strongly correlated with aging [5, 6]. ASE alone strongly inhibited the irradiation-induced increases of MMP-1 *in vitro* (Figure 3(a)). Moreover, rather than suppressing the activity of retinoids in this model, ASE actually enhanced their ability to inhibit MMP-1 expression (Figure 3(b)). Thus, ASE may not only directly inhibit MMP-1 expression, but also indirectly promotes the skin antiaging effects, boosting of retinoid action. 

Afferent somatic nerves with unmyelinated (C-) or myelinated (A*δ*-) fibers innervate the skin, and they respond to a range of chemicals and physiologic stimuli such as heat, cold, nociception, and UV light, participating in cutaneous inflammation. On stimulation, the nerves rapidly release active neuropeptides such as CGRP, tachykinins, and vasoactive intestinal peptide (VIP), then they act on target cells resulting in erythema, edema, hyperthermia, and pruritus associated with sensitive skin symptoms [49]. In the present study, we measured CGRP expression level after capsaicin treatment in neuronal cells [50] then identified whether ASE works as a potential inhibitor of CGRP-induced neurogenic inflammation. Capsaicin might trigger cytosolic Ca^2+^ influx through delta opioid receptor (DOR) activation in human SK-N-BE(2) and SH-SY5Y neuroblastoma cells [51–53], and elevated Ca^2+^ levels could induce CGRP release from neuronal cells [54]. Our present results also revealed that ASE decreased the release of CGRP from SK-N-BE(2) and SH-SY5Y neuroblastoma cells following capsaicin treatment in a dose-dependent manner (Table 1) [55]. This suggests that ASE may reduce sensitive skin symptoms related to cutaneous neurogenic inflammation. 

## 5. Conclusions

We herein show that ASE contained echitamine and loganin as its major compounds and could potentially be used as an anti-irritation agent to counter unwanted skin symptoms such as those induced by retinoid treatment. ASE not only markedly decreased several components of retinoid-induced dermatitis, it but also boosted the ability of retinoids to inhibit MMP-1 protein expression, suggesting that it could enhance the antiwrinkle effects of retinoids. We are currently examining the molecular basis for this enhancement effect, but the present study provides evidence suggesting that ASE should be considered a good candidate for development as a biologically effective anti-irritation compound that is also capable of conferring antiwrinkle effects.

## Figures and Tables

**Table 1:** Anti-inflammatory effect of *Alstonia scholaris* extract in *all-trans* retinoic acid treated HaCaT human keratinocytes.

Test substances	MCP-1		IL-8	
	pg/mL	% of inhibition	pg/mL	% of inhibition
ASE 500 ppm + ATRA 1 *μ*M	928.8 ± 64.0**	96.6	417.1 ± 29.0**	83.9
ASE 100 ppm + ATRA 1 *μ*M	1074.0 ± 82.2**	82.1	619.3 ± 44.4**	26.3

Madecassoside 500 ppm + ATRA 1 uM	1271.0 ± 69.0*	62.5	589.8 ± 31.0*	34.7
Madecassoside 100 ppm + ATRA 1 uM	1221.6 ± 100.8*	67.4	584.6 ± 70.6**	36.2
Hydrocortisone 1 ppm + ATRA 1 *μ*M	1027.6 ± 48.5**	86.8	407.7 ± 20.8*	86.6

ATRA 1 *μ*M	1898.2 ± 72.0	0	711.8 ± 27.0	0
Control	894.7 ± 141.0	100.0	360.4 ± 28.8	100.0

ASE: *Alstonia scholaris* extract; ATRA: *all-trans* retinoic acid; MCP-1: monocyte chemoattractant protein-1; IL-8: interleukin-8; Results are expressed as mean ± SD. *P < 0.05 and **P < 0.01 compared with ATRA-treated group values.

**Table:** (a)

Test conditions	ng/mL	% of inhibition
ASE 0.1 ppm + UVB 40 mJ	621.8 ± 27.8**	59.4
ASE 1 ppm + UVB 40 mJ	451.0 ± 38.4**	98.8
ASE 10 ppm + UVB 40 mJ	331.2 ± 22.2**	126.5

ATRA 1 *μ*M + UVB 40 mJ	557.4 ± 9.7**	74.3
ATRA 5 *μ*M + UVB 40 mJ	505.3 ± 64.1*	86.3

ROL 1 *μ*M + UVB 40 mJ	708.9 ± 30.8*	39.2
ROL 5 *μ*M + UVB 40 mJ	574.9 ± 51.1*	70.2

UVB 40 mJ	878.5 ± 30.2	0
Control	446.0 ± 28.5	100.0

MMP-1: Matrix metalloproteinase-1; ASE: *Alstonia scholaris* extract; ATRA: *all-trans* retinoic acid; ROL: retinol. *P < 0.05 and **P < 0.01 compared with UVB-irradiated group values.

**Table:** (b)

Test substance	ng/mL	% of inhibition
ATRA 1 *μ*M + UVB 40 mJ	557.4 ± 9.7**	74.3
ASE 0.1 ppm + ATRA 1 *μ*M + UVB 40 mJ	424.6 ± 6.9**	105.0
ASE 1 ppm + ATRA 1 *μ*M + UVB 40 mJ	379.7 ± 18.3**	115.3
ASE 10 ppm + ATRA 1 *μ*M + UVB 40 mJ	335.6 ± 50.5**	125.5

ROL 1 *μ*M + UVB 40 mJ	708.9 ± 30.8*	39.2
ASE 0.1 ppm + ROL 1 *μ*M + UVB 40 mJ	522.5 ± 13.3**	82.3
ASE 1 ppm + ROL 1 *μ*M + UVB 40 mJ	439.4 ± 38.4**	101.5
ASE 10 ppm + ROL 1 *μ*M + UVB 40 mJ	389.3 ± 28.1**	113.1

UVB 40 mJ	878.5 ± 30.2	0.0
Control	446.0 ± 28.5	100.0

MMP-1: Matrix metalloproteinase-1; ASE: *Alstonia scholaris* extract; ATRA: *all-trans* retinoic acid; ROL: retinol. *P < 0.05 and **P < 0.01 compared with UVB-irradiated group values.

**Table 3:** Evaluation of capsaicin-induced CGRP expression in the presence or absence of ASE in the SH-SY5Y and SK-N-BE(2) neuroblastoma cell lines.

Test substance	SH-SY5Y		SK-N-BE(2)	
	pg/mL	% of inhibition	pg/mL	% of inhibition
Capsaicin 300 nM + ASE 1 ppm	27.2 ± 2.9*	97.7	5.3 ± 1.3	79.0
Capsaicin 300 nM + ASE 10 ppm	24.6 ± 3.8*	104.0	4.6 ± 0.2*	108.4
Capsaicin 300 nM	66.6 ± 11.9	0	7.2 ± 0.8	0
Control	26.3 ± 2.1	100	4.8 ± 1.2	100

*P < 0.05 compared with capsaicin-treated group values.

**Table 4:** Visual scoring results. Test emulsion contained with 0.1% ASE with ROL 2500 IU; control: control emulsion with ROL 2500 IU.

Subject	0.1% ASE + ROL 2500 IU	Control (ROL 2500 IU)
#1	3	3
#2	4	4
#3	4	5
#4	1	2
#5	0	2
#6	0	0
#7	1	2
#8	0	4
#9	4	12
#10	0	7
#11	1	3
#12	2	3
#13	0	1
#14	6	6
#15	0	17
#16	5	12
#17	5	8
#18	0	10
#19	4	10
#20	1	0
#21	0	1
Sum**^†^**	**41**	**112**
Mean**^‡^**	1.95*	**5.33**

^†^means as sum value of the daily irritation scores of all subjects for a test compound. A maximum score was 1260 (21 persons × 15 days × 4).

^‡^calculated as follows: mean = sum/n (the number of subjects; *n* = 21).

*P < 0.01 was calculated versus control (retinol treated).
